# Dipotassium tetra­aqua­bis­[3,5-bis­(dicyano­methyl­ene)cyclo­pentane-1,2,4-trionato(1−)-κ*N*]cobaltate(II)

**DOI:** 10.1107/S1600536810048646

**Published:** 2010-11-27

**Authors:** Luciano Honorato Chagas, Jan Janczak, Flavia C. Machado, Luiz Fernando C. de Oliveira, Renata Diniz

**Affiliations:** a Núcleo de Espectroscopia e Estrutura Molecular (NEEM), Department of Chemistry, Federal University of Juiz de Fora – Minas Gerais, 36036-900, Brazil; b Institute of Low Temperature and Structure Research, Polish Academy of Sciences, Wroclaw, PO Box 1410 50-950, Poland

## Abstract

The title structure, K_2_[Co(C_11_N_4_O_3_)_2_(H_2_O)_4_], is isotypic with K_2_[Fe(C_11_N_4_O_3_)_2_(H_2_O)_4_]. The Co^II^ atom is in a distorted octa­hedral CoN_2_O_4_ geometry, forming a dianionic mononuclear entity. Each dianionic unit is associated with two potassium cations and inter­acts with adjacent units through O—H⋯N and O—H⋯O hydrogen bonds.

## Related literature

For the structure and applications of the croconate violet dianion [3,5-bis-(dicyano­methyl­ene)cyclo­pentane-1,2,4-trionate], see: Fatiadi (1978 ▶); Dumestre *et al.* (1998 ▶); Teles *et al.* (2006 ▶); De Abreu *et al.* (2009 ▶); Faria *et al.* (2010 ▶); Garcia *et al.* (2010 ▶). For the synthesis and applications of pseudo-oxocarbons, see: West & Niu (1963 ▶), Fatiadi (1980 ▶); Galibert *et al.* (2001 ▶); De Oliveira *et al.* (2009 ▶). For the isotypic compound, K_2_[Fe(C_11_N_4_O_3_)_2_(H_2_O)_4_], see: Soula *et al.* (2003 ▶).
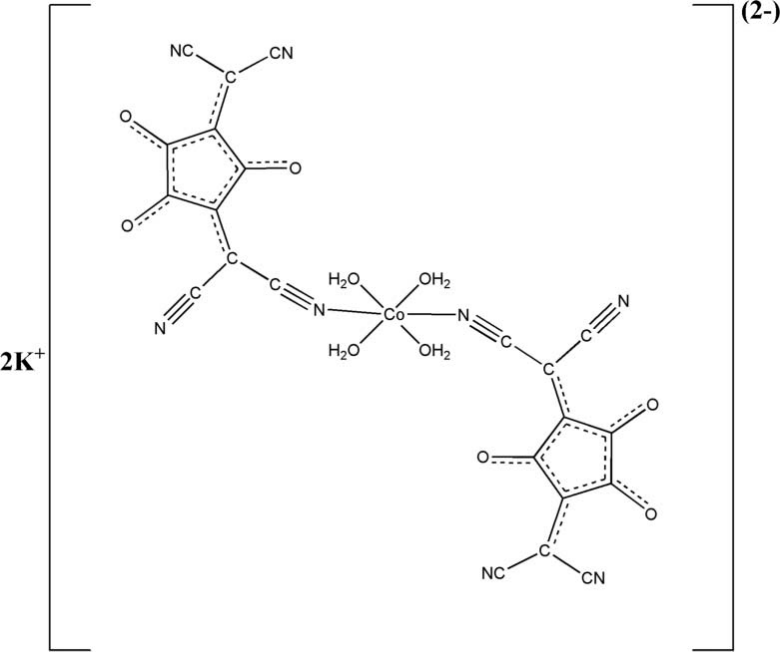


## Experimental

### 

#### Crystal data

K_2_[Co(C_11_N_4_O_3_)_2_(H_2_O)_4_]
*M* *_r_* = 681.49
Monoclinic, 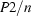

*a* = 9.4060 (19) Å
*b* = 7.0110 (14) Å
*c* = 19.493 (4) Å
β = 92.58 (3)°
*V* = 1284.2 (5) Å^3^
*Z* = 2
Mo *K*α radiation
μ = 1.07 mm^−1^
*T* = 293 K
0.26 × 0.24 × 0.16 mm

#### Data collection

Kuma KM-4-CCD diffractometer
Absorption correction: analytical (*SHELXTL*; heldrick, 2008 ▶) *T*_min_ = 0.765, *T*_max_ = 0.843
15657 measured reflections
3181 independent reflections
1839 reflections with *I* > 2σ(*I*)
*R*_int_ = 0.037

#### Refinement

*R*[*F* ^2^ > 2σ(*F* ^2^)] = 0.033
*wR*(*F* ^2^) = 0.095
*S* = 0.91
3181 reflections
195 parameters
H-atom parameters constrained
Δρ_max_ = 0.20 e Å^−3^
Δρ_min_ = −0.18 e Å^−3^

### 

*KM-4-CCD Software* (Kuma, 2004 ▶); cell refinement: *KM-4-CCD Software*; data reduction: *KM-4-CCD Software*; program(s) used to solve structure: *SHELXS97* (Sheldrick, 2008 ▶); program(s) used to refine structure: *SHELXL97* (Sheldrick, 2008 ▶); molecular graphics: *ORTEP-3 for Windows* (Farrugia, 1997 ▶); software used to prepare material for publication: *PLATON* (Spek, 2009 ▶).

## Supplementary Material

Crystal structure: contains datablocks global, I. DOI: 10.1107/S1600536810048646/bt5409sup1.cif

Structure factors: contains datablocks I. DOI: 10.1107/S1600536810048646/bt5409Isup2.hkl

Additional supplementary materials:  crystallographic information; 3D view; checkCIF report

## Figures and Tables

**Table 1:** Hydrogen-bond geometry (Å, °)

*D*—H⋯*A*	*D*—H	H⋯*A*	*D*⋯*A*	*D*—H⋯*A*
O1—H1⋯O2^i^	0.97	1.86	2.785 (3)	159
O1—H2⋯N2^ii^	0.96	1.92	2.880 (3)	177
O4—H3⋯N4^iii^	0.97	2.23	2.779 (3)	115
O4—H4⋯O2^iv^	0.97	1.73	2.681 (3)	167

Symmetry codes: (i) 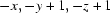; (ii) 
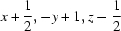
; (iii) 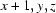; (iv) 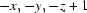.

**Fig. 1.**
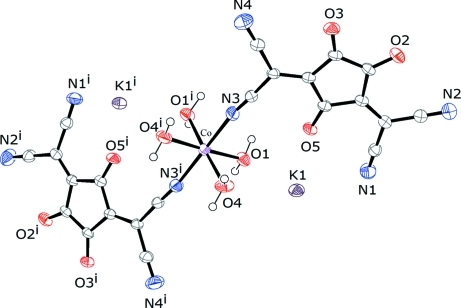
The molecular structure of K2[Co(CV)2(H2O)4] showing 50% displacement ellipsoids. Symmetry code: i -x + 1/2, y, -z + 1/2.

**Fig. 2.**
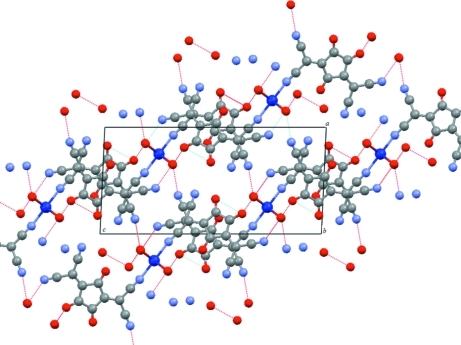
View of the contribution of hydrogen bonds for stacking along of ab plane.

**Fig. 3.**
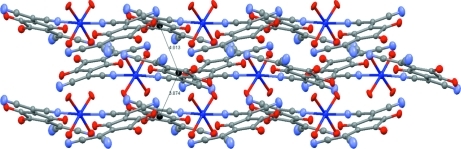
View of the crystal packing of K2[Co(CV)2(H2O)4] along of ab plane, evidencing centroid-centroid distances between adjacent sheets. Hydrogen and potassium atoms were omitted for clarity.

## supplementary crystallographic information

### Comment

The pseudo-oxocarbons are derived of oxocarbons which are cyclic planar species of general formula (C_n_O_n_)^2-^ where *n* varies from 3 to 6 (West & Niu, 1963). In the pseudo-oxocarbons one or more oxygen atoms are replaced by other atoms or groups (Fatiadi, 1978). This class of compounds has received great attention due to various possible coordination modes (De Oliveira *et al.*, 2009). Specially, the dianion Croconate Violet (CV) [3,5-bis-(dicyanomethylene)cyclopentane-1,2,4-trionate], which is displayed in Scheme I, plays the important role from the structural and spectroscopic viewpoints due to extensive π delocalization and electrical conductivity typical of semiconductor materials (Teles *et al.*, 2006).

The structure related in this report is isostructural to previously described by Soula *et al.* (2003) but this time the central metal ion is cobalt (II) instead of iron (II) (Scheme II). Crystal structure of K_2_[Co(CV)_2_(H_2_O)_4_] is depicted in Figure 1. The metallic ion is surrounded octahedrically by four oxygen atoms from the water molecules and two nitrogen atoms from two different CV units forming a dianionic mononuclear discrete entity which is neutralized by two potassium cations that act as counter ions. Each CV is coordinated in monodentate way to the metal site. The cobalt atom sits in a special position on twofold axes. As commonly observed for cobalt (II) complexes, for the compound under study there is a distortion of octahedral geometry evidenced by two Co—O1 distances (2.1348 (18) Å) longer than Co—O4 and Co—N3 (2.075 (18) Å and 2.088 (2) Å, respectively). For the free nitrogen atoms (N1, N2 and N4) is observed that the CN triple bond lengths vary from 1.131 (3) to 1.137 (3) Å. For the coordinated nitrogen (N3) this distance is 1.153 (3) Å. The ring C—C and C—O bond lengths vary to 1.441 (3) to 1.476 (3) Å and 1.227 (3) to 1.246 (3) Å, respectively, confirming the π electron delocalization over the pseudo-oxocarbon ring (Teles *et al.*, 2006).

Besides the interactions with potassium ions the compound has intermolecular hydrogen interactions in which the oxygen atom of water molecule (O1) interact with atoms of oxygen (O2) and nitrogen (N2) of adjacent CV. Moreover, moderate hydrogen bonds occur between O4 (of coordinated water) and N4 and O2 (of Croconate Violet). Hydrogen bonds contribute to the crystal packing extending the chain along the crystallographic directions a and b (Fig.2). Centroid-centroid distances are around 3.9 (2) and 4.0 (2) Å (Fig.3) and the calculated interplanar distances are around 3.3 (6) Å.

### Experimental

The pseudo-oxocarbon Croconate Violet was obtained according to method described in literature (Teles *et al.*, 2006). The original intention was to obtain a bimetallic compound. In this sense we proceeded as follow: 0.70 g (2.5 mmol) of potassium salt of Croconate Violet (K_2_CV 2.5H_2_O) was dissolved in 32 ml of a 1:1/(acetonitrile:water) at room temperature. This solution was added to 25 ml of aqueous solution of CoCl_2_.6H_2_O (0.60 g, 2.5 mmol). On this mixture was added, slowly, 15 ml of aqueous solution containing FeSO_4_.7H_2_O (0.70 g, 2.5 mmol). Good crystals suitable to *X* ray diffraction were obtained after one month and characterized by *X* ray diffraction as K_2_[Fe(CV)_2_(H_2_O)_4_]. The solution was filtered and set aside for crystallization by slow evaporation of the solvents. Two week later violet single crystals with metallic luster, suitable to *X* ray diffraction, were obtained and characterized as K_2_[Co(CV)_2_(H_2_O)_4_]. Elemental analysis calculated for C_22_O_10_N_8_H_8_K_2_Co: C (38.77); H (1.18); N (16.44). Found: C (37.21); H (1.23); N (15.16).

FT—IR analysis: 1468 cm^-1^ (νCC + νCO), 1520 cm^-1^ (νCO + νCC(CN)_2_), 1605 cm^-1^ (ν_ass_CO), 1682 cm^-1^ (νCO), 2214 cm^-1^ (νCN), 2246 cm^-1^ (_coordinated_νCN), 3368 cm^-1^ (νOH).

Raman spectroscopy (room temperature, 632.8 nm) corroborate with FT—IR analyses and permits attribute bands characteristics of CV coordinated by only one nitrogen atom: 1498 cm^-1^ (νCC + νCO), 1583 cm^-1^ (νCO + νCC(CN)_2_), 1604 cm^-1^ (ν_ass_CO), 1686 cm^-1^ (νCO), 2220 cm^-1^ (νCN), 2242 cm^-1^ (_coordinated_νCN).

### Refinement

H atoms  were located from electron density maps, fixed in these positions and assigned the same isotropic displacement parameters for all H atoms.

### Crystal data

**Table:**

K_2_[Co(C_11_N_4_O_3_)_2_(H_2_O)_4_]	*F*(000) = 683.80
*M**_r_* = 681.49	*D*_x_ = 1.762 Mg m^−^^3^
Monoclinic, *P*2/*n*	Mo *K*α radiation, λ = 0.71073 Å
Hall symbol: -P 2yac	Cell parameters from 1839 reflections
*a* = 9.4060 (19) Å	θ = 2.9–29.0°
*b* = 7.0110 (14) Å	µ = 1.07 mm^−^^1^
*c* = 19.493 (4) Å	*T* = 293 K
β = 92.58 (3)°	Prism, violet
*V* = 1284.2 (5) Å^3^	0.26 × 0.24 × 0.16 mm
*Z* = 2	

### Data collection

**Table:**

Kuma KM-4-CCD diffractometer	3181 independent reflections
Radiation source: fine-focus sealed tube	1839 reflections with *I* > 2σ(*I*)
graphite	*R*_int_ = 0.037
CCD scans	θ_max_ = 29.0°, θ_min_ = 2.9°
Absorption correction: analytical (*SHELXTL*; Sheldrick, 2008)	*h* = −12→12
*T*_min_ = 0.765, *T*_max_ = 0.843	*k* = −6→9
15657 measured reflections	*l* = −26→26

### Refinement

**Table:**

Refinement on *F*^2^	H-atom parameters constrained
Least-squares matrix: full	*w* = 1/[σ^2^(*F*_o_^2^) + (0.0521*P*)^2^] where *P* = (*F*_o_^2^ + 2*F*_c_^2^)/3
*R*[*F*^2^ > 2σ(*F*^2^)] = 0.033	(Δ/σ)_max_ < 0.001
*wR*(*F*^2^) = 0.095	Δρ_max_ = 0.20 e Å^−^^3^
*S* = 0.91	Δρ_min_ = −0.18 e Å^−^^3^
3181 reflections	Extinction correction: *SHELXL97* (Sheldrick, 2008)
195 parameters	Absolute structure: no
0 restraints	

### Special details

**Table:**

Geometry. All e.s.d.'s (except the e.s.d. in the dihedral angle between two l.s. planes) are estimated using the full covariance matrix. The cell e.s.d.'s are taken into account individually in the estimation of e.s.d.'s in distances, angles and torsion angles; correlations between e.s.d.'s in cell parameters are only used when they are defined by crystal symmetry. An approximate (isotropic) treatment of cell e.s.d.'s is used for estimating e.s.d.'s involving l.s. planes.
Refinement. Refinement of *F*^2^ against ALL reflections. The weighted *R*-factor *wR* and goodness of fit *S* are based on *F*^2^, conventional *R*-factors *R* are based on *F*, with *F* set to zero for negative *F*^2^. The threshold expression of *F*^2^ > σ(*F*^2^) is used only for calculating *R*-factors(gt) *etc*. and is not relevant to the choice of reflections for refinement. *R*-factors based on *F*^2^ are statistically about twice as large as those based on *F*, and *R*- factors based on ALL data will be even larger.

### Fractional atomic coordinates and isotropic or equivalent isotropic displacement parameters (Å^2^)

**Table:**

	*x*	*y*	*z*	*U*_iso_*/*U*_eq_	
Co1	0.2500	0.17828 (7)	0.2500	0.02924 (16)	
K1	0.42443 (6)	0.23207 (9)	0.44538 (3)	0.03741 (19)	
C1	0.0167 (3)	0.3045 (3)	0.56951 (13)	0.0236 (6)	
C2	−0.1309 (3)	0.2706 (3)	0.58237 (13)	0.0252 (6)	
O2	−0.18964 (19)	0.3004 (2)	0.63736 (9)	0.0364 (5)	
C3	−0.2022 (3)	0.1925 (3)	0.51963 (13)	0.0249 (6)	
O3	−0.32681 (18)	0.1369 (3)	0.51461 (9)	0.0354 (5)	
C4	−0.0990 (2)	0.1952 (3)	0.46673 (13)	0.0228 (6)	
C5	0.0376 (3)	0.2594 (3)	0.49667 (13)	0.0244 (6)	
O5	0.14928 (18)	0.2729 (3)	0.46684 (9)	0.0342 (5)	
C6	0.1215 (3)	0.3657 (3)	0.61569 (13)	0.0273 (6)	
C7	−0.1203 (2)	0.1460 (3)	0.39800 (13)	0.0250 (6)	
C8	0.2638 (3)	0.4102 (4)	0.59492 (13)	0.0298 (6)	
N1	0.3752 (2)	0.4503 (4)	0.57986 (12)	0.0433 (6)	
C9	0.0935 (3)	0.3955 (4)	0.68581 (15)	0.0341 (7)	
N2	0.0713 (3)	0.4206 (4)	0.74186 (14)	0.0553 (7)	
C10	−0.0119 (3)	0.1587 (4)	0.35161 (13)	0.0265 (6)	
N3	0.0736 (2)	0.1691 (3)	0.31124 (11)	0.0352 (6)	
C11	−0.2557 (3)	0.0854 (4)	0.36967 (14)	0.0313 (6)	
N4	−0.3609 (3)	0.0374 (4)	0.34506 (13)	0.0539 (7)	
O1	0.34625 (18)	0.3966 (3)	0.31272 (9)	0.0346 (5)	
H1	0.2763	0.4939	0.3210	0.052*	
H2	0.4233	0.4533	0.2891	0.052*	
O4	0.35063 (17)	−0.0188 (3)	0.31486 (10)	0.0409 (5)	
H3	0.4359	−0.0690	0.2954	0.061*	
H4	0.2877	−0.1230	0.3255	0.061*	

### Atomic displacement parameters (Å^2^)

**Table:**

	*U*^11^	*U*^22^	*U*^33^	*U*^12^	*U*^13^	*U*^23^
Co1	0.0229 (3)	0.0403 (3)	0.0250 (3)	0.000	0.0053 (2)	0.000
K1	0.0232 (3)	0.0488 (4)	0.0404 (4)	0.0034 (3)	0.0033 (3)	−0.0022 (3)
C1	0.0221 (12)	0.0186 (13)	0.0302 (14)	0.0025 (11)	0.0045 (11)	0.0011 (10)
C2	0.0236 (13)	0.0237 (14)	0.0291 (14)	0.0030 (11)	0.0097 (11)	0.0011 (11)
O2	0.0331 (10)	0.0412 (12)	0.0361 (11)	−0.0011 (9)	0.0153 (9)	−0.0018 (9)
C3	0.0184 (12)	0.0253 (14)	0.0313 (14)	0.0067 (12)	0.0051 (11)	0.0013 (11)
O3	0.0200 (9)	0.0480 (12)	0.0386 (11)	0.0059 (9)	0.0048 (8)	−0.0034 (8)
C4	0.0200 (12)	0.0178 (13)	0.0308 (14)	0.0026 (11)	0.0028 (11)	0.0010 (10)
C5	0.0226 (13)	0.0205 (13)	0.0305 (14)	0.0021 (11)	0.0043 (11)	0.0014 (11)
O5	0.0191 (9)	0.0507 (13)	0.0336 (10)	−0.0026 (9)	0.0091 (8)	−0.0030 (8)
C6	0.0269 (14)	0.0255 (15)	0.0297 (15)	−0.0003 (11)	0.0046 (12)	0.0007 (11)
C7	0.0171 (12)	0.0294 (15)	0.0287 (14)	0.0008 (11)	0.0031 (11)	0.0011 (11)
C8	0.0314 (15)	0.0283 (15)	0.0295 (15)	−0.0027 (12)	−0.0019 (12)	−0.0019 (13)
N1	0.0320 (14)	0.0540 (16)	0.0443 (15)	−0.0039 (13)	0.0045 (12)	−0.0128 (12)
C9	0.0271 (15)	0.0432 (17)	0.0320 (16)	−0.0033 (14)	0.0013 (12)	0.0026 (13)
N2	0.0454 (16)	0.084 (2)	0.0370 (16)	−0.0075 (15)	0.0020 (13)	0.0111 (15)
C10	0.0229 (13)	0.0291 (15)	0.0273 (14)	−0.0014 (12)	−0.0004 (11)	−0.0007 (11)
N3	0.0287 (13)	0.0471 (15)	0.0300 (13)	−0.0039 (11)	0.0030 (11)	−0.0023 (11)
C11	0.0238 (14)	0.0362 (16)	0.0341 (15)	−0.0003 (13)	0.0040 (12)	−0.0004 (13)
N4	0.0330 (14)	0.070 (2)	0.0584 (18)	−0.0073 (15)	−0.0049 (13)	−0.0078 (14)
O1	0.0308 (10)	0.0376 (11)	0.0363 (11)	−0.0030 (9)	0.0102 (8)	−0.0030 (8)
O4	0.0234 (10)	0.0456 (12)	0.0541 (13)	0.0165 (10)	0.0065 (9)	−0.0017 (9)

### Geometric parameters (Å, °)

**Table:**

Co1—O4	2.0725 (18)	C3—C4	1.448 (3)
Co1—O4	2.0725 (18)	O3—K1^iv^	2.731 (2)
Co1—N3	2.088 (2)	O3—K1^ii^	2.8647 (19)
Co1—N3	2.088 (2)	C4—C7	1.389 (3)
Co1—O1	2.1348 (18)	C4—C5	1.458 (3)
Co1—O1	2.1348 (18)	C5—O5	1.227 (3)
K1—O5	2.6557 (18)	C6—C9	1.419 (4)
K1—O3^i^	2.731 (2)	C6—C8	1.449 (3)
K1—O3^ii^	2.865 (2)	C7—C10	1.396 (3)
K1—O1	2.896 (2)	C7—C11	1.429 (3)
K1—N1^iii^	2.972 (2)	C8—N1	1.137 (3)
K1—N1	3.088 (2)	N1—K1^iii^	2.972 (2)
K1—O4	3.145 (2)	C9—N2	1.135 (3)
K1—N4^i^	3.181 (3)	C10—N3	1.153 (3)
C1—C6	1.374 (4)	C11—N4	1.131 (3)
C1—C2	1.441 (3)	N4—K1^iv^	3.181 (3)
C1—C5	1.476 (3)	O1—H1	0.9666
C2—O2	1.246 (3)	O1—H2	0.9616
C2—C3	1.474 (4)	O4—H3	0.9683
C3—O3	1.235 (3)	O4—H4	0.9689
			
O1···O4	2.913 (3)	O4···O1	2.913 (3)
O1···N3	3.019 (3)	O4···N3^v^	2.906 (3)
O1···O1^v^	2.976 (3)	O4···O2^ii^	2.681 (3)
O1···N3^v^	3.019 (3)	O5···N1	3.240 (3)
O1···O2^vi^	2.785 (3)	O5···N3	3.169 (3)
O1···N2^vii^	2.880 (3)	N1···O5	3.240 (3)
O2···O4^ii^	2.681 (3)	N2···O2	3.230 (3)
O2···O3	2.903 (3)	N2···O1^viii^	2.880 (3)
O2···N2	3.230 (3)	N3···O5	3.169 (3)
O2···O1^vi^	2.785 (3)	N3···O1	3.019 (3)
O3···O2	2.903 (3)	N3···O4	2.918 (3)
O4···O4^v^	3.090 (3)	N3···O4^v^	2.906 (3)
O4···N4^i^	2.779 (3)	N3···O1^v^	3.019 (3)
O4···N3	2.918 (3)	N4···O4^iv^	2.779 (3)
			
O4—Co1—O4	96.39 (11)	O4—K1—K1^iii^	154.16 (4)
O4—Co1—N3	89.06 (8)	N4—K1^i^—K1^iii^	35.60 (3)
N3—Co1—N3	176.46 (13)	K1—K1^ix^—K1^iii^	37.61 (3)
O4—Co1—O1	87.62 (8)	C6—C1—C2	127.3 (2)
N3—Co1—O1	91.27 (8)	C6—C1—C5	125.1 (2)
O4—Co1—O1	87.62 (8)	C2—C1—C5	107.6 (2)
N3—Co1—O1	91.27 (8)	O2—C2—C1	126.2 (2)
O1—Co1—O1	88.39 (10)	O2—C2—C3	125.0 (2)
O5—K1—O3^i^	140.17 (6)	C1—C2—C3	108.8 (2)
O5—K1—O3^ii^	74.21 (5)	O3—C3—C4	127.7 (2)
O3—K1^i^—O3^ii^	11.69 (3)	O3—C3—C2	125.3 (2)
O5—K1—O1	83.55 (6)	C4—C3—C2	106.9 (2)
O3—K1^i^—O1	20.96 (3)	C3—O3—K1^iv^	138.66 (16)
O3—K1^ii^—O1	38.27 (5)	C3—O3—K1^ii^	125.34 (16)
O5—K1—N1^iii^	125.09 (7)	K1—O3^iv^—K1^ii^	21.60 (3)
O3—K1^i^—N1^iii^	20.56 (4)	C7—C4—C3	127.7 (2)
O3—K1^ii^—N1^iii^	39.77 (5)	C7—C4—C5	123.3 (2)
O1—K1—N1^iii^	72.02 (6)	C3—C4—C5	109.0 (2)
O5—K1—N1	68.19 (6)	O5—C5—C4	126.3 (2)
O3—K1^i^—N1	14.67 (3)	O5—C5—C1	126.4 (2)
O3—K1^ii^—N1	41.41 (5)	C4—C5—C1	107.4 (2)
O1—K1—N1	121.26 (6)	C5—O5—K1	158.07 (17)
N1—K1^iii^—N1	83.66 (7)	C1—C6—C9	121.2 (2)
O5—K1—O4	90.38 (6)	C1—C6—C8	121.9 (2)
O3—K1^i^—O4	20.22 (3)	C9—C6—C8	116.8 (2)
O3—K1^ii^—O4	55.19 (5)	C4—C7—C10	122.1 (2)
O1—K1—O4	57.48 (5)	C4—C7—C11	122.3 (2)
N1—K1^iii^—O4	52.18 (6)	C10—C7—C11	115.6 (2)
N1—K1—O4	158.10 (6)	N1—C8—C6	177.8 (3)
O5—K1—N4^i^	142.49 (7)	C8—N1—K1^iii^	145.8 (2)
O3—K1^i^—N4^i^	21.06 (4)	C8—N1—K1	105.78 (19)
O3—K1^ii^—N4^i^	56.13 (5)	K1—N1^iii^—K1	96.34 (7)
O1—K1—N4^i^	76.39 (6)	N2—C9—C6	179.5 (3)
N1—K1^iii^—N4^i^	66.21 (6)	N3—C10—C7	177.3 (3)
N1—K1—N4^i^	149.08 (7)	C10—N3—Co1	171.5 (2)
O4—K1—N4^i^	52.11 (6)	N4—C11—C7	177.6 (3)
O5—K1—K1^ix^	108.71 (5)	C11—N4—K1^iv^	100.3 (2)
O3—K1^i^—K1^ix^	21.37 (3)	Co1—O1—K1	108.05 (7)
O3—K1^ii^—K1^ix^	70.56 (5)	Co1—O1—H1	109.1
O1—K1—K1^ix^	147.83 (5)	K1—O1—H1	106.2
N1—K1^iii^—K1^ix^	41.49 (6)	Co1—O1—H2	109.4
N1—K1—K1^ix^	90.81 (5)	K1—O1—H2	115.1
O4—K1—K1^ix^	92.01 (4)	H1—O1—H2	108.8
N4—K1^i^—K1^ix^	26.62 (3)	Co1—O4—K1	101.47 (7)
O5—K1—K1^iii^	97.14 (5)	Co1—O4—H3	111.6
O3—K1^i^—K1^iii^	28.27 (3)	K1—O4—H3	111.3
O3—K1^ii^—K1^iii^	36.25 (5)	Co1—O4—H4	111.5
O1—K1—K1^iii^	98.72 (4)	K1—O4—H4	111.3
N1—K1^iii^—K1^iii^	42.81 (5)	H3—O4—H4	109.5
N1—K1—K1^iii^	40.85 (5)		
			
O4—Co1—O1—K1	−20.82 (8)	N1—K1—O3^ii^—C3^ii^	86.9 (2)
N3—Co1—O1—K1	68.19 (8)	K1^iv^—O3—C3—C4	−64.1 (4)
O1^v^—Co1—O1—K1	159.42 (8)	K1^iv^—O3—C3—C2	115.6 (3)
N3^v^—Co1—O1—K1	−109.35 (8)	K1^ii^—O3—C3—C2	−82.7 (3)
O5—K1—O1—Co1	−78.47 (8)	K1^ii^—O3—C3—C4	97.6 (3)
N1—K1—O1—Co1	−138.07 (7)	K1—O5—C5—C1	47.5 (6)
O3^i^—K1—O1—Co1	109.16 (10)	K1—O5—C5—C4	−132.2 (4)
N1^iii^—K1—O1—Co1	151.36 (9)	C2—C1—C5—C4	−1.0 (2)
O3^ii^—K1—O1—Co1	−11.68 (10)	C5—C1—C2—O2	−176.9 (2)
O1—K1—O5—C5	174.6 (5)	C2—C1—C6—C8	−174.6 (2)
N1—K1—O5—C5	−58.0 (5)	C6—C1—C5—O5	−1.3 (4)
O3^i^—K1—O5—C5	−13.7 (5)	C6—C1—C5—C4	178.5 (2)
N1^iii^—K1—O5—C5	−122.7 (5)	C5—C1—C6—C9	−176.4 (2)
O3^x^—K1—O5—C5	47.9 (5)	C2—C1—C5—O5	179.3 (2)
O1—K1—N1—C8	89.2 (2)	C2—C1—C6—C9	2.9 (4)
O1—K1—N1—K1^iii^	−64.49 (8)	C5—C1—C6—C8	6.1 (4)
O5—K1—N1—C8	21.78 (19)	C6—C1—C2—C3	−175.8 (2)
O5—K1—N1—K1^iii^	−131.87 (8)	C6—C1—C2—O2	3.7 (4)
O3^xi^—K1—N1—C8	−131.4 (2)	C5—C1—C2—C3	3.7 (2)
O3^i^—K1—N1—K1^iii^	74.97 (7)	C1—C2—C3—O3	175.3 (2)
N1—K1—N1—C8	153.7 (2)	C1—C2—C3—C4	−4.9 (2)
N1^xii^—K1—N1—K1^xii^	0.02 (11)	O2—C2—C3—O3	−4.2 (4)
O3^ii^—K1—N1—C8	−47.2 (2)	O2—C2—C3—C4	175.6 (2)
O3^ii^—K1—N1—K1^iii^	159.17 (6)	O3—C3—C4—C7	4.0 (4)
O1—K1—O3^i^—C3^i^	32.0 (3)	O3—C3—C4—C5	−175.9 (2)
O5—K1—O3^i^—C3^i^	−136.1 (2)	C2—C3—C4—C5	4.3 (2)
N1—K1—O3^i^—C3^i^	−95.3 (3)	C2—C3—C4—C7	−175.8 (2)
O1—K1—N1^iii^—K1^iii^	125.80 (7)	C3—C4—C5—O5	177.6 (2)
O1—K1—N1^iii^—C8^iii^	−103.7 (4)	C7—C4—C5—C1	178.0 (2)
O5—K1—N1^iii^—K1^iii^	57.67 (9)	C3—C4—C7—C10	178.6 (2)
O5—K1—N1^iii^—C8^iii^	−171.8 (3)	C3—C4—C7—C11	0.8 (4)
N1—K1—N1^xii^—K1^xii^	0.00 (7)	C5—C4—C7—C10	−1.5 (3)
N1—K1—N1^iii^—C8^iii^	130.6 (4)	C5—C4—C7—C11	−179.3 (2)
O1—K1—O3^ii^—C3^ii^	−49.0 (2)	C3—C4—C5—C1	−2.1 (2)
O5—K1—O3^ii^—C3^ii^	22.69 (19)	C7—C4—C5—O5	−2.3 (4)

Symmetry codes: (i) *x*+1, *y*, *z*; (ii) −*x*, −*y*, −*z*+1; (iii) −*x*+1, −*y*+1, −*z*+1; (iv) *x*−1, *y*, *z*; (v) −*x*+1/2, *y*, −*z*+1/2; (vi) −*x*, −*y*+1, −*z*+1; (vii) *x*+1/2, −*y*+1, *z*−1/2; (viii) *x*−1/2, −*y*+1, *z*+1/2; (ix) −*x*+1, −*y*, −*z*+1; (x) −*x*+1, −*y*−1, −*z*+1; (xi) *x*+1, *y*−1, *z*−1;  (xii) −*x*, −*y*+1, −*z*.

### Hydrogen-bond geometry (Å, °)

**Table:**

*D*—H···*A*	*D*—H	H···*A*	*D*···*A*	*D*—H···*A*
O1—H1···O2^vi^	0.97	1.86	2.785 (3)	159
O1—H2···N2^vii^	0.96	1.92	2.880 (3)	177
O4—H3···N4^i^	0.97	2.23	2.779 (3)	115
O4—H4···O2^ii^	0.97	1.73	2.681 (3)	167

Symmetry codes: (vi) −*x*, −*y*+1, −*z*+1; (vii) *x*+1/2, −*y*+1, *z*−1/2; (i) *x*+1, *y*, *z*; (ii) −*x*, −*y*, −*z*+1.
